# Can the Shape of a Planar Pathway Be Estimated Using Proximal Forces of Inserting a Flexible Shaft?

**DOI:** 10.3389/frobt.2021.757895

**Published:** 2021-11-02

**Authors:** Jiajun Liu, Lin Cao, Soo Jay Phee

**Affiliations:** ^1^ Robotics Research Centre, School of Mechanical and Aerospace Engineering, Nanyang Technological University, Singapore, Singapore; ^2^ Department of Automatic Control and Systems Engineering, The University of Sheffield, Sheffield, United Kingdom

**Keywords:** shape, insertion, force, neural network model, flexible rod

## Abstract

The shape information of flexible endoscopes or other continuum structures, e.g., intro-vascular catheters, is needed for accurate navigation, motion compensation, and haptic feedback in robotic surgical systems. Existing methods rely on optical fiber sensors, electromagnetic sensors, or expensive medical imaging modalities such as X-ray fluoroscopy, magnetic resonance imaging, and ultrasound to obtain the shape information of these flexible medical devices. Here, we propose to estimate the shape/curvature of a continuum structure by measuring the force required to insert a flexible shaft into the internal channel/pathway of the continuum. We found that there is a consistent correlation between the measured insertion force and curvature of the planar continuum pathway. A testbed was built to insert a flexible shaft into a planar continuum pathway with adjustable shapes. The insertion forces, insertion displacement, and the shapes of the pathway were recorded. A neural network model was developed to model this correlation based on the training data collected on the testbed. The trained model, tested on the testing data, can accurately estimate the curvature magnitudes and the accumulated bending angles of the pathway simply based on the measured insertion force at the proximal end of the shaft. The approach may be used to estimate the curvature magnitudes and accumulated bending angles of flexible endoscopic surgical robots or catheters for accurate motion compensation, haptic force feedback, localization, or navigation. The advantage of this approach is that the employed proximal force can be easily obtained outside the pathway or continuum structure without any embedded sensor in the continuum structure. Future work is needed to further investigate the correlation between insertion forces and the pathway and enhance the capability of the model in estimating more complex shapes, e.g., spatial shapes with multiple bends.

## Introduction

Obtaining the shape information of continuum structures or manipulators, e.g., flexible endoscopes and intra-vascular catheters, is desirable in navigation, motion control and compensation, and haptic feedback. During colonoscopy, due to the lack of the shape information of the colonscope, loop formation occurs frequently and often causes patient discomfort, prolonged procedure, and risk of perforations (Cheng et al., 2013). For flexible endoscopic surgical robots (Cao et al., 2019; Cao et al., 2020) and intra-vascular catheters (Khoshnam and Patel 2016; Hu et al., 2018), the shape of the endoscope/catheter has a significant effect on the force and motion transmission of the system. The friction loss of the force transmission system is dependent on the accumulated bending angle of the endoscope/catheter, and the elongation of the driving cables even depends on how the accumulated angle or curvature changes along the pathway (Sun et al., 2015).

Existing methods for the shape measurement/prediction of continuum manipulators include 1) intraoperative imaging (Chen et al., 2017; Janjic et al., 2018) (magnetic resonance imaging, ultrasound, and X-ray, etc.), 2) sensing (Khoshnam and Patel 2016; Song et al., 2018; Zhuang et al., 2018) (electro-magnetic tracking, Fiber Bragg Grating (FBG) arrays, etc.), and 3) modeling based on kinematics and mechanics (Xu and Simaan 2010; Chirikjian 2015).

Intraoperative imaging is widely adopted in the clinic, but it is often done with bulky and expensive medical imaging modalities, and the patients may be exposed to radiation (e.g., X-ray). *In-situ* sensors can provide rich, accurate, and real-time shape information but require reliable sensor integration (sealing, sterilization, etc.) with the system and increases the size and cost of the system.

Many studies (Khatait et al., 2012; Back et al., 2015; Khoshnam et al., 2015; Cardoso and Furuie 2016; Hu et al., 2019) have attempted to compute the kinematics and kinetics (motion, insertion forces, and contact forces, etc.) of a flexible shaft in a pathway with a specified shape. The insertion force when inserting a flexible rod into a frictionless rigid and zero-clearance channel could be modeled by $\frac{EI\kappa^{2}}{2}$ (Dal Corso et al., 2017), showing a quadratic relation between the curvature of the channel and the required insertion force. However, with clearance and friction, the external forces become more complex even in simple situations (Chen and Li 2007; Liu and Chen 2013). The nonlinear relations between insertion force and the channel shape could be described using a set of ODEs with the integral of variables satisfying various constraints. This is an integral constraint problem, which is difficult to solve analytically or inverse mathematically.

With the emerging of data driven methods, it is possible to obtain the probability-weighted correlation between two data sets. Neural Network (NN), inspired by biological neural cells and neural networks, is widely used in natural language processing, image processing, and visual recognition problems. Multilayer perceptron (MLP), Recurrent-Neural-Networks (RNN) and Convolutional Neural Networks (CNN) are classic architectures of NN. MLP is suitable for classification problems. RNN is designed to handle sequence prediction problems. CNN shows its dominating advantages on problem involving image data as an input. NN could be constructed by stacking MLP, RNN, and CNN. The Long-short-term-memory (LSTM) is the most successful RNN, which is wildly used in various engineering studies.

Li and Cao (2019), Li et al. (2019) constructed a hybrid model involving multiple NN model constructed by CNN LSTM and MLP to predict the force transmitted along a tendon sheath mechanism of arbitrary accumulated angle. Zhao et al. (2017) trained a CNN-Deep bidirectional LSTM-MLP achieving better prediction in signal process comparing with pure LSTM network.

The insertion process is about feeding a flexible shaft into the hollow channel of a continuum structure with a constant velocity. In this work, we propose to train a neural network to capture the correlation between insertion force and channel’s curvature (or shape). The trained neural network model could predict the shape/curvature of the channel based on the insertion force measured.

**FIGURE 1:**
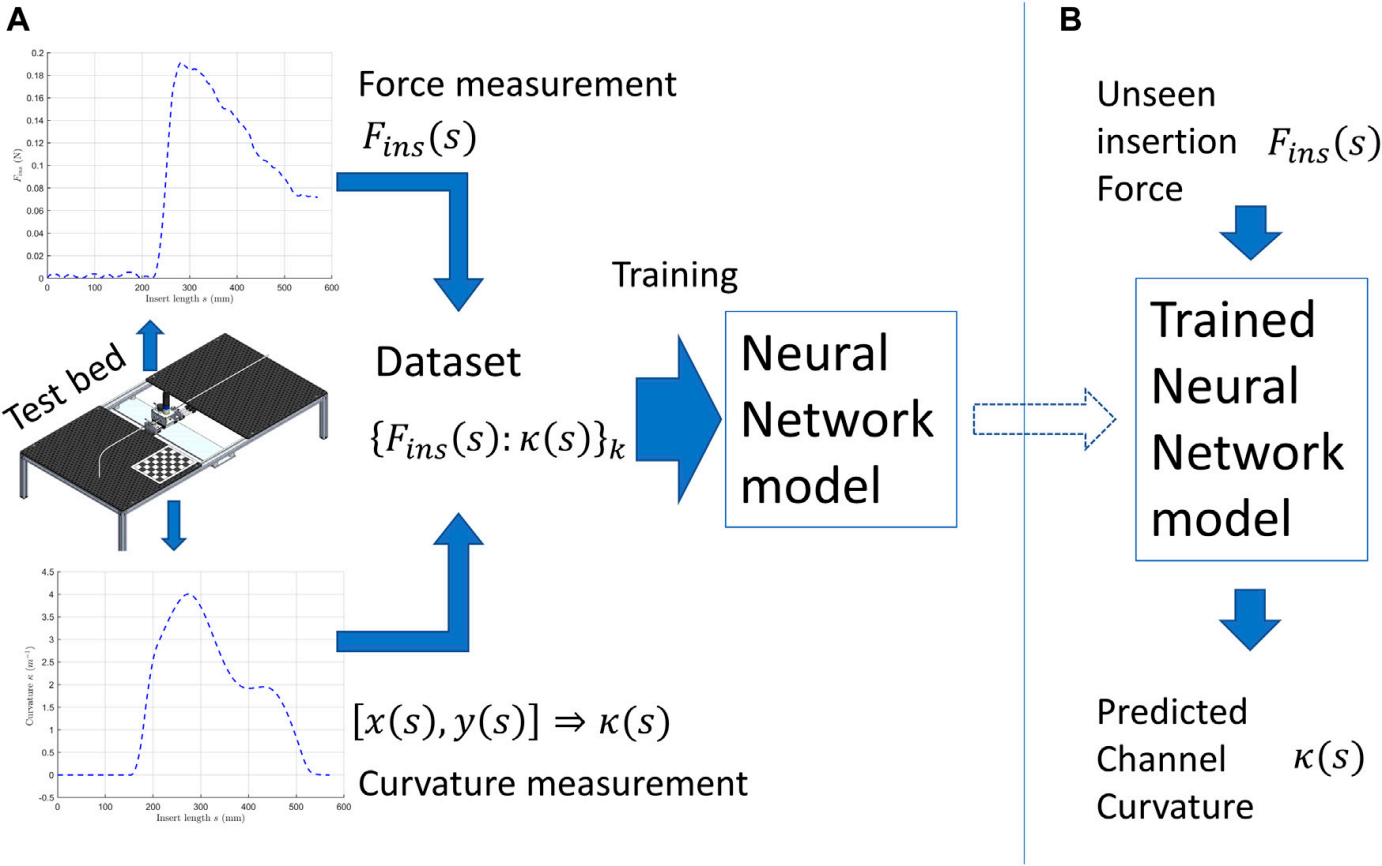
Illustration of the proposed method. **(A)** data collection and model training. **(B)** using the model to predict curvature.

Figure 1 illustrates the main idea of this work. In Figure 1A, we developed a testbed that can insert a flexible nitinol shaft into the channel (a tube fixed on the testbed). The insertion force $F_{ins}\left(s\right)$ is measured by loadcells on platform. The insertion length s is recorded by the encoder. Then, the shape of the tube can be described the curvature κ(s) along its length, which is obtained from the top-view image. The insertion force $F_{ins}\left(s\right)$ and curvature κ(s) forms the data for training. As shown in Figure 1B, the trained model can predict the curvature κ(s) of the tube based on the insertion force Fins(s). Then, the coordinates of the tube [x(s),y(s)] could be obtained from the predicted curvature κ(s) .

The paper is organized as follows. In *Experimental Setup and Data Collection*, the force and curvature measurements and data processing are introduced. *The Neural Network Model* presents the proposed neural network model. Results and discussions are presented in *Results and Discussion*. Finally, *Conclusion* concludes this research.

## Experimental Setup and Data Collection

This work aims to investigate how the shape information of a continuum pathway can be predicted based on simply the measured force required to insert a flexible shaft. We built a testbed that can insert a flexible shaft into a shape-adjustable tube/pathway for data collection (insertion forces, insertion displacement, and tube shapes). The collected data will be used for 1) to verify the correlation between shapes and insertion forces, 2) to train and test and the proposed neural network model. As an initial attempt, we only focus on 2D shapes, and the shapes have only two-bend shapes at most. This section introduces the experimental setup, data collection, and data processing approaches.

### Experimental Setup

**FIGURE 2:**
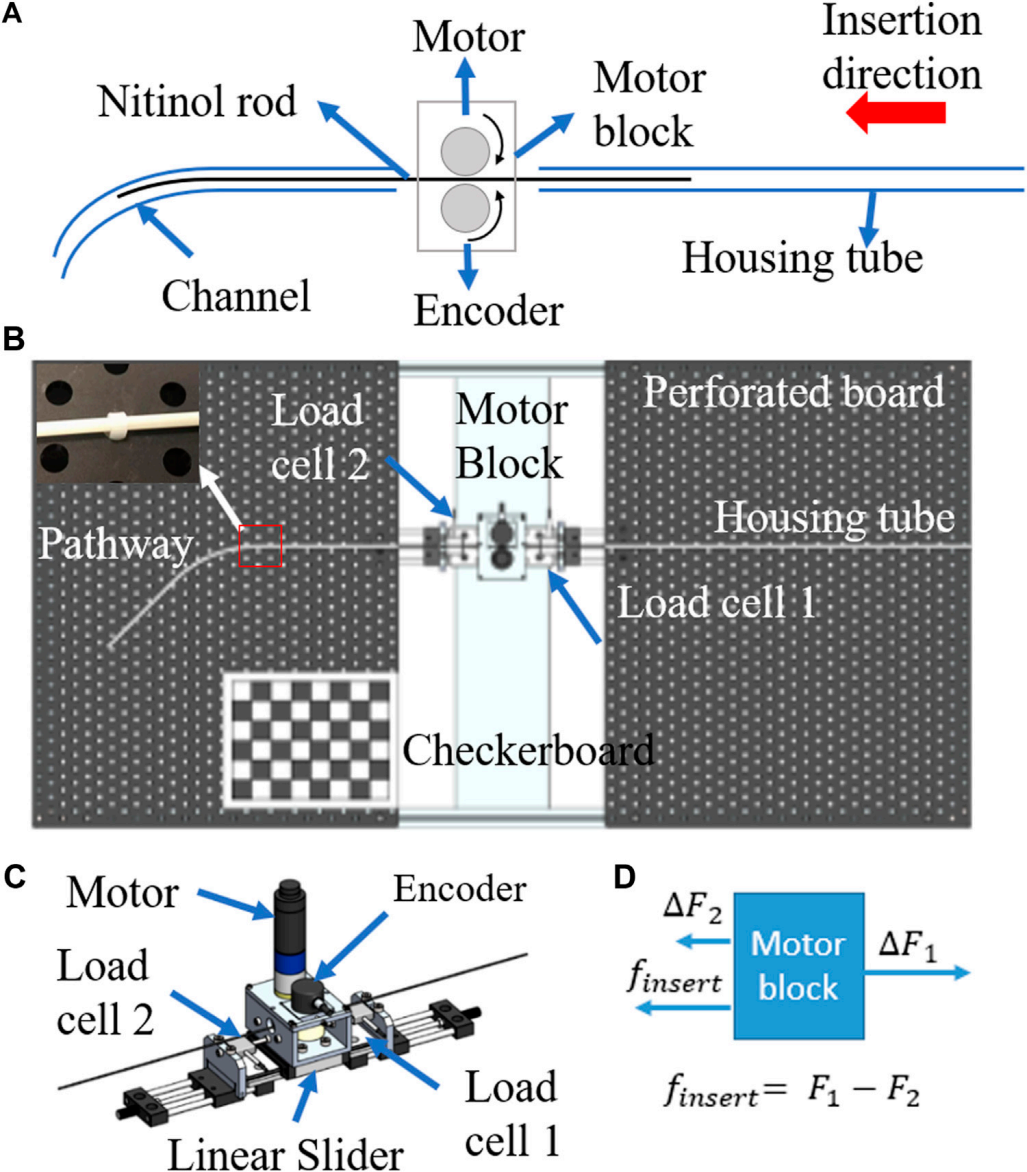
**(A)** schematic diagram of experimental setup, **(B)** experimental setup, **(C)** the design of the motor block, **(D)** force diagram of the motor block.

Figure 2 shows the schematic diagram of the experimental setup. In Figure 2A, the motor block drove the flexible shaft (a super-elastic nitinol tube with OD/ID: 1.27/0.97mm, NI207130, GOODFELLOW®) into the curved channel (glass fiber reinforced plastic tube OD/ID: 3/1.5 mm). The motor (2342S024C, FAULHABER®) and the encoder (2RM3600-D, SCANCON®, 3,600 pulse/rev) clamped the flexible shaft by two rubber rollers of 13.5 mm diameter. A housing tube was used to support the remaining nitinol tube outside the motor block. It also reduced the vibration of the nitinol tube. The overview of the testbed is shown in Figure 2B. The glass fiber reinforced plastic tube (channel) was bent into desired shapes and fixed on to the perforated board. The top-view image of the curved channel with the checkerboard was taken. The checkerboard was used as a reference when calculating the curvature along the channel. In Figure 2C, the motor block was mounted on a linear slider (SSEBWZ16-110, MISUMI®). Two loadcells (FUTEK® LSB200, capacity 4.5 N, rated output 0.1 mV/V) sandwiched the motor block were fixed on two linear sliders. Tuning these linear sliders could adjust the pretension applied on the motor block.

### Measuring Insertion Force

MATLAB SIMULINK interfaced with a QPIDe data acquisition board from Quanser Quarc® was used to control the motor and acquire and synchronize sensor readings. The motor was controlled to insert the flexible shaft into the channel at a speed of 20.0 mm/s. Once insertion length s = 700 mm was reached, the motor would rest for 5 seconds and then withdraw the shaft to the starting point. The motor rested for 5 seconds again and this process was repeated two times.

A force diagram is provided in Figure 2D. The insertion force required is denoted as $\;F_{ins}\;$ . Both load cells are pre-loaded to $\;F_{0}\;$ before measurement. The load cell readings are denoted as $F_{1}$ and $F_{2}$ . The forces from load cell 1 to the motor block is $\;\Delta F_{1}=F_{1}-F_{0}$ , and the force from load cell 2 is $\;\Delta F_{2}=F_{2}-F_{0}$ . The total insertion force is as follows:

$$\;F_{ins}=\;\Delta F_{1}-\;\Delta F_{2}=F_{1}-F_{2}$$

**FIGURE 3:**
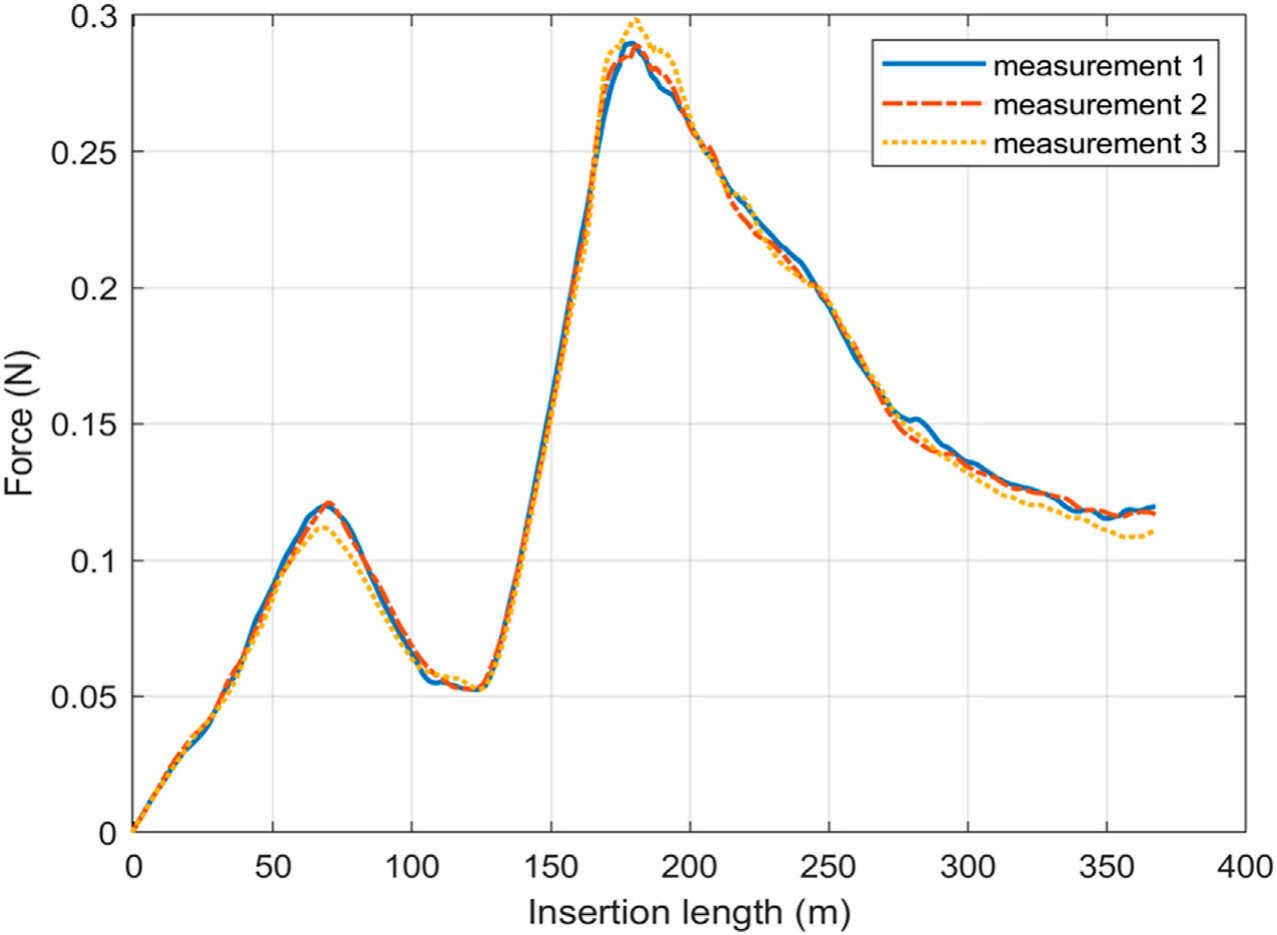
The repeated measurements of the insertion force on a channel has the two-bend shape.

A second-order low-pass filter (cutoff frequency, sampling rate) was applied for both load cells. Data recorded from insertion distances 30–600 mm was used for this study. Then, the insertion length L = 570 mm and s∈[0,570] mm. In this range, the motor block was in its most stable condition and the shaft is driven with constant speed. After calibration of all load cells, the platform was used to collect data. Figure 3 shows three repeated measurements on a channel, and the maximum deviation is 3% comparing with the mean value. It indicates the insertion force for a given shape is consistent, and the measurement system is reliable. Note that the frictions of the housing tube and linear bearing are neglectable.

### Measuring Channel Curvature

The top-view image of the channel and the checkerboard was taken. The point clouds of the channel could be obtained via imaging processing in terms of the pixels. These pixels could be converted to real-world coordinates with respect to the checkerboard. Then, a parameter curve could be fitted to the point clouds and then used to calculate coordinates, curvatures, and accumulated bending angles.

**FIGURE 4:**
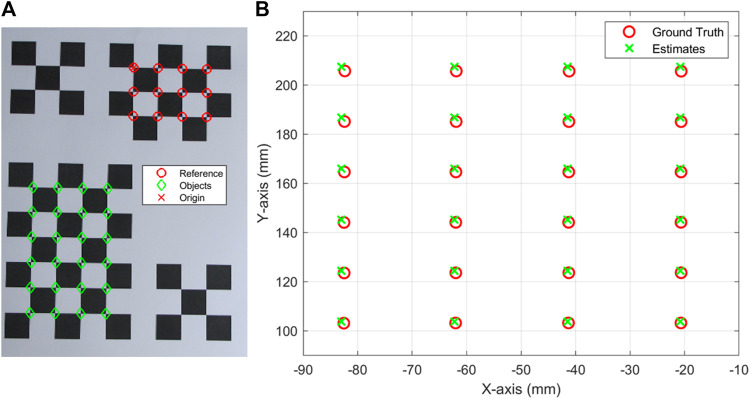
**(A)** checkerboards to check the accuracy of image measurement, **(B)** the ground truth and estimates of the objects in **(A)**.

First, the camera parameters were obtained using the checkerboard fixed on the platform using MATLAB camera calibration function “estimateCameraParameters”. Then, the object in the image could be measured using the checkerboard and camera parameters. To illustrate the accuracy of this measurement, two checkerboards were placed diagonally on one A4 paper, as shown in Figure 4A. One checkerboard was the reference checkerboard, and the other was the targeting/object checkerboard. Figure 4B shows the estimations and ground truth of the objects. The average and maximum distances between the estimates and ground truth are 1.26 and 1.88 mm, which are small enough for this application.

**FIGURE 5:**
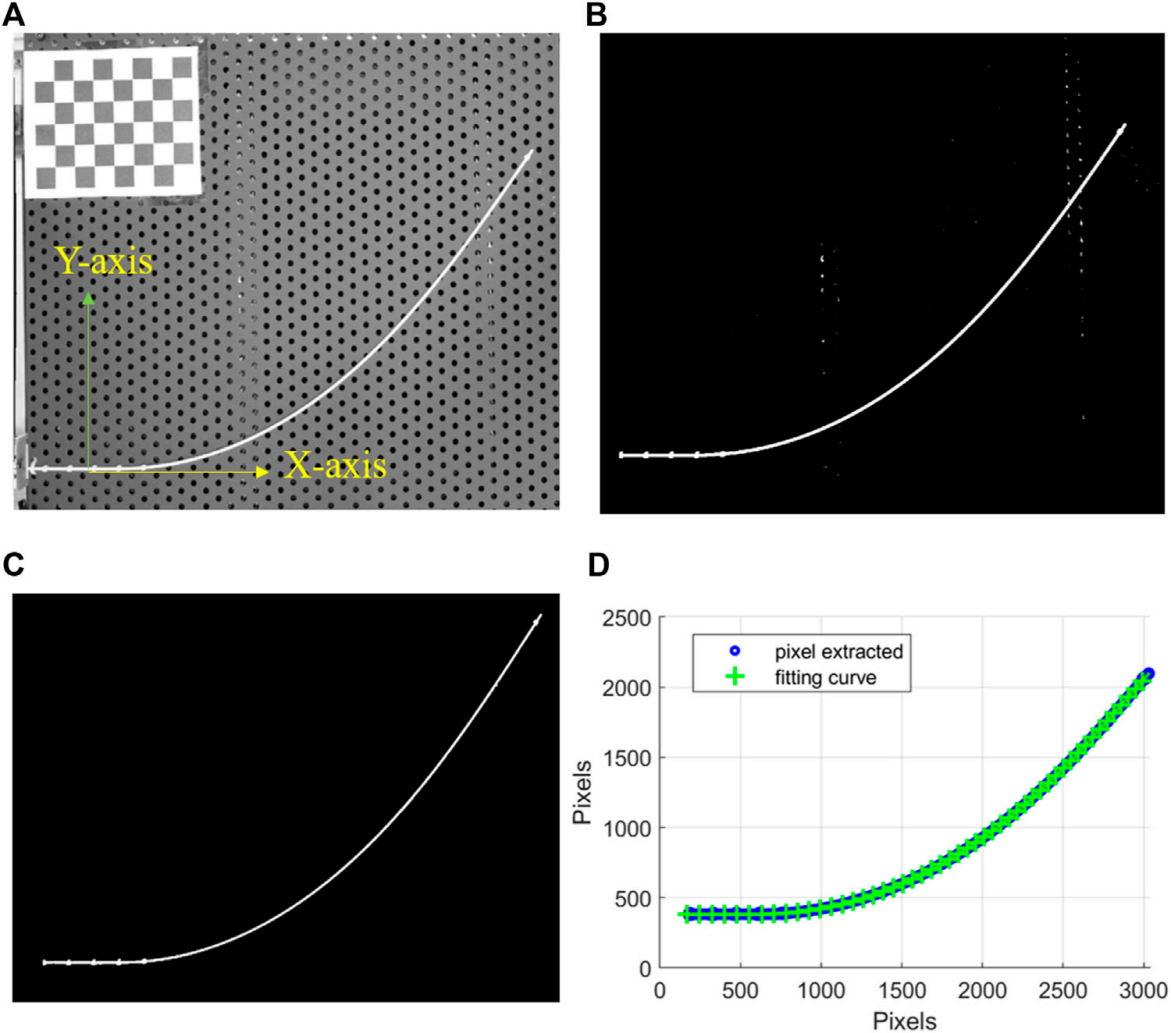
**(A)** Greyscale image, and coordination setup, **(B)** binary image with noise, **(C)** binary image with noise removed, **(D)** point clouds from the binary image and fitting curve in the image frame.

Then, we took the image of the channel during the insertion experiment. These images were post-processed to obtain the channel shape. Figure 5 shows the steps of extracting the channel coordinates with respect to the checkerboard from images taken. First, the fisheye effect (lens distortion) was removed using the camera parameters. Then, as shown in Figures 5A, a greyscale image was obtained by saturating the top and bottom 1% values of the green channel of the image. Then the greyscale image was converted to a binary image with a threshold of 180 as Figure 5B. Then the binary image would be converted to point clouds in coordinates form. The noises were removed from the tube objects, resulting in a clean background in Figure 5C. The point clouds were fitted with 1) straight lines for the beginning and ending 2) ninth-order polynomial for the curved section. A fifth-order spline interpolation replaced the intersection zone of linear fittings and the polynomial fitting to obtain a smooth curvature profile as shown in Figure 5C. The starting point of the insertion shaft is set to be the origin. Figure 5D shows the point cloud obtained and the fitting curve. We obtained the acutal shape $P_{channel}=\left[x,y\right]$ by converting these coordinates in the pixel to mm using the checkerboard. The origin of the acutal shape $P_{channel}$ was fixed as shown in Figure 5A. All negative x points of the curve were discarded. The insertion length of m^th^ point in the curve is calculated as the length of the fitting curve from the original point to the m^th^ point:

$$s_{m}=\{\begin{array}{c} \;\;\;\;\;\;\;\;\;\;\;\;\;\;\;\;\;\;\;\;\;\;\;\;\;\;\;\;\;\;\;\;\;\;\;\;\;\;\;\;\;\;0\;\;\;\;\;\;\;\;\;\;\;\;\;\;\;\;\;\;\;\;\;\;\;\;\;\;\;\;\;\;\;\;\;\;\;\;\;\;\;\;\;\;\;\;\;\;\;for\;m=1\\ \overset{m}{\underset{i=2}{\sum }}\sqrt{{\left(x_{i}-x_{i-1}\right)}^{2}+{\left(y_{i}-y_{i-1}\;\right)}^{2}}\;\;\;\;\;\;\;\;\;\;\;\;\;\;for\;m>1 \end{array}$$

Then curvature was calculated based on the fitting curve using LineCurvature2D (Kroon 2021). The incremental angle for each point is calculated by

$$\begin{array}{l} d\theta_{i}=\{\begin{array}{c} \;\;\;\;\;\;\;\;\;\;\;\;\;\;\;\;\;\;\;\;\;\;\;\;\;\;\;\;\;\;\;\;\;0\;\;\;\;\;\;\;\;\;\;\;\;\;\;\;\;\;\;\;\;\;\;\;\;\;\;\;\;\;for\;i=1\;or\;i=n\\ \tan^{-1}\frac{\;\Delta y_{i+1}}{\Delta x_{i+1}}-\tan^{-1}\frac{\;\Delta y_{i}}{\Delta x_{i}}\;\;\;\;\;\;\;\;\;fori\neq 1\;and\;i\neq n \end{array}\\ where\;\Delta x_{i}=\;x_{i}-x_{i-1}\;and\;\Delta y_{i}=\;y_{i}-y_{i-1}\;\; \end{array}$$

The accumulated bending angle from beginning to m^th^ point is calculated by:

$$\theta_{m}=\;\sum_{i=0}^{n}\;||d\theta_{i}||\;.$$

### Overview of Data Collected

**FIGURE 6:**
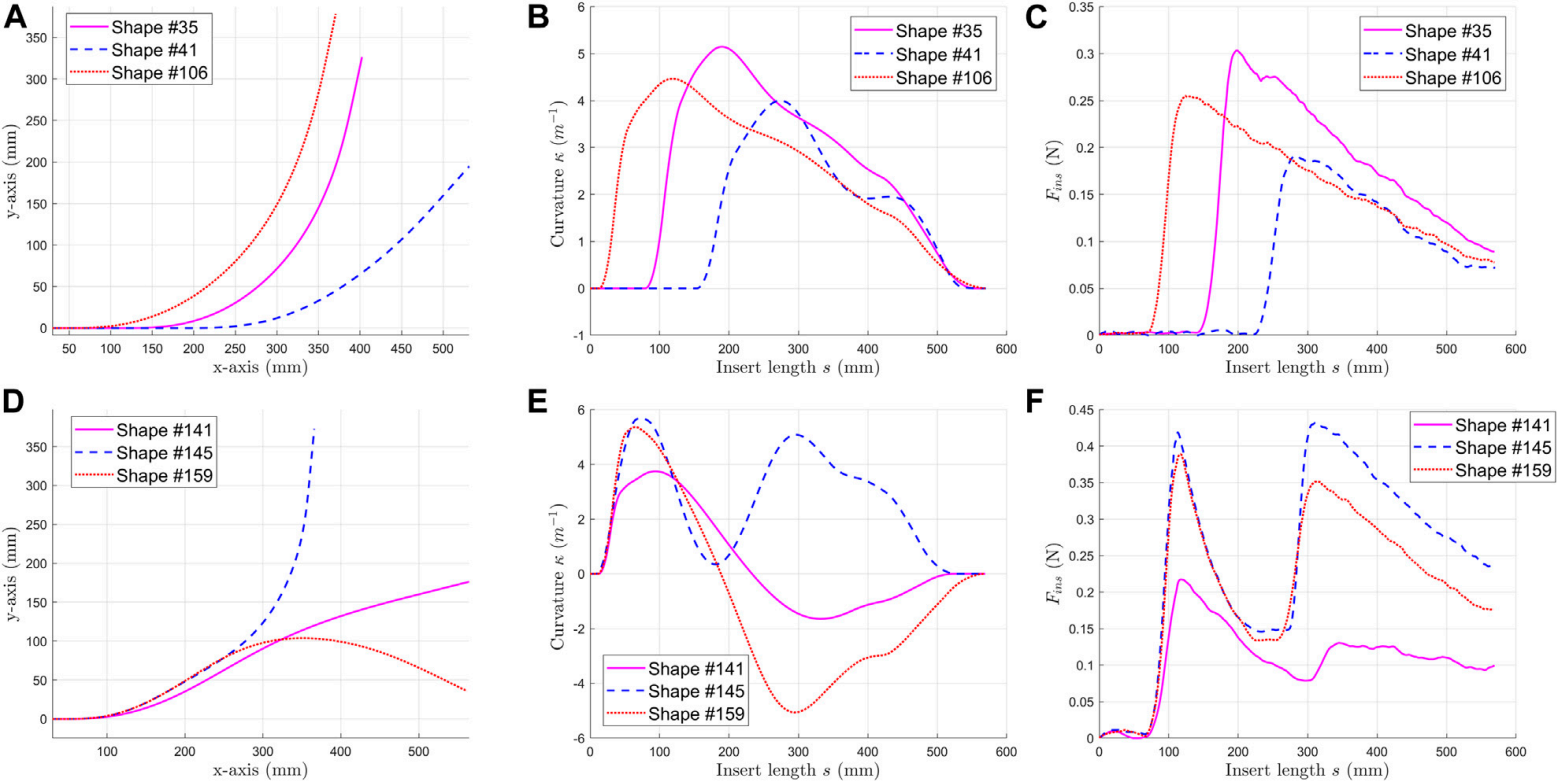
Some examples of data collected. For one-bend shape #35, #41j: **(A)** coordinates of the channel shapes, **(B)** curvature of the channel shapes, **(C)** insertion forces of the selected shapes. For one-bend shape #141, #145Ÿ: **(D)** coordinates of the channel shapes, **(E)** curvature of the channel shapes, **(F)** insertion forces of the selected shapes.

The insertion forces were measured when inserting the shaft into the curved channels. The channel curvatures were measured from the top-view image. The insertion force and respective channel curvatures were grouped as one set of data. By changing the shape of the channel, different insertion forces were obtained. As summarized in Table 1, we collected the channel curvatures and insertion forces of 120 one-bend shapes (indexed from #1 to #120) and 39 two-bend shapes (indexed from #121 to #159). One-bend shapes #35, #41& #106 in Figure 6A and two-bend shapes #141, #145 & #159 in Figure 6B are shown as an example The ranges of curvature and accumulated angle are also listed in Table 1. Moreover, the curvature was set to be positive for one-bend shapes. For two-bend shapes, the first bend had positive curvature, but the second bend could be positive or negative. The insertion force profiles and curvatures for one-bend shapes are shown in Figures 6B,C and those for two-bend shapes are shown in Figures 6E,F. It can be observed that the insertion force changes in a nonlinear manner in Figure 6C as the curvature changes in Figure 6B. Then, these shapes were grouped into two different datasets for the neural network model as Table 2. We used 105 one-bend shapes as training/dev set and 15 one-bend shapes as test set. Dataset 2 mixed one-bend shape and two-bend shapes. All one-bend shapes and 32 two-bend shapes were in training/dev set while 7 two-bend shapes were in test set.

**TABLE 1:** The channel shapes with different features.

No.	Starting location (mm)	No. of bends	Accumulated angle (degree)		Curvatures (1/m)		No. shapes
			Min	Max	Min	Max	
1	30	1	0.68	87.08	0.04	4.95	62
2	80	1	3.69	84.63	0.32	5.44	31
3	130	1	6.99	84.28	0.54	6.83	27
4	40	2	20.22	90.26	−5.06	5.84	39

**TABLE 2:** The compositions of the datasets.

Dataset	Train and Dev	Test
1	105 one-bend shapes (#1, 2, 3)	15 one-bend shapes
2	120 one-bend shapes (#1, 2, 3) 32 two-bend shapes (#4)	7 two-bend shapes

## The Neural Network Model

### Structure of the NN

Then, we used the data driven method to investigate association between the insertion force profile and the channel shape.

First, the data obtained (insertion force profile and channel shape) are sequential. At any time, the curvature along the channel could be treated as function of insert length, provided the clearance is small. Then we also record the insertion force profile with respect to insertion length. The sequential data could be handled by the long-short-term-memory (LSTM) network. The bi-directional LSTM is formed by stacking two LSTM of opposite directions. The bi-directional LSTM has advantages in processing the context of a sequential data.

**FIGURE 7:**
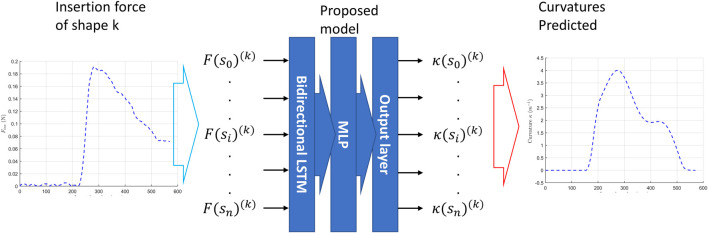
An illustration of the proposed neural network model.

The structure of the proposed deep neural network model is shown in Figure 7. The network has an input as: $F_{ins}{\left(s_{i}\right)}^{\left(k\right)}\;\;$ at every insertion length step *i* for shape *k*. The label or output is the curvatures $\;\kappa {\left(s_{i}\right)}^{\left(k\right)}\;$ at insertion length step *i* for shape *k*. The input data was first fed to the bi-directional LSTM layers and then output through a MLP network. A simple way of enhancing the capability of a LSTM network is to increase the number of layers. However, if only LSTM is used, as the number of layers increases, more variables inside the LSTM need to be handled, and the training time will significantly increase. Therefore, multiple bidirectional LSTM layers are used for feature extraction and multilayer perceptron (MLP) is stacked to handle the information from bidirectional LSTM, which has been proved its advantages in (Zhao et al., 2017).

### Model Parameters and System Information

The network parameters were eventually selected using Sklearn. gridsearchCV: four layers of bidirectional LSTM, two layers of MLP with dropout layers in between, 120 hidden neurons for all layers. The dropout ratio was fixed at 0.5. Adam as optimizer and Huber loss-function are the best pick of parameters for the proposed neural network. The training stops at 5,000 epochs, where loss has converged. The configuration of the workstation: processor: Inter® Core (TM) i5-7500; RAM: 16G; GPU: Nvidia GTX 1050Ti. The CuDNNLSTM (Keras.layers) was used instead of normal LSTM layers to reduce training times. With parameters listed above, 5-fold cross validation was performed, i.e., the proposed neural network was trained and tested on five splits of Dataset 1. The training scores and testing scores for each split dataset were calculated. Finally, it is to check whether the network fits all the split datasets equally.

## Results and Discussion

The trained model outputted the curvature $\kappa_{p}\left(s\right)$ , which can be used to calculate the coordinates of the channel $p_{channel}=\left[x_{p}\left(s\right),y_{p}\left(s\right)\right]$ .

$$x_{p}\left(s\right)=\;\overset{s}{\underset{0}{\int }}\text{cos}\left(\theta \left(s\right)\right)ds+x\left(0\right)$$

$$y_{p}\left(s\right)=\;\overset{s}{\underset{0}{\int }}\text{sin}\left(\theta \left(s\right)\right)ds+y\left(0\right)$$

$$\theta \left(s\right)=\;\overset{s}{\underset{0}{\int }}\kappa_{p}\left(s\right)ds+0$$

**FIGURE 8:**
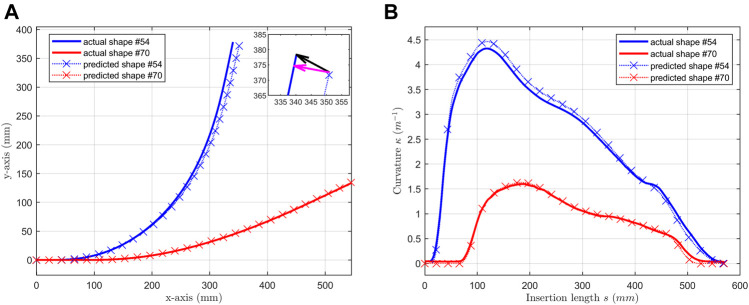
Selected shapes (shape #54 & #70) predicted by the NN model with bidirectional LSTM. **(A)** predicted shapes are compared with actual shapes; **(B)** predicted curvatures are compared with actual curvatures.

The error, which is the difference between the predicted shape $p_{channel}\;$ and the actual channel shape $P_{channel}$ , is used to evaluate the performance of trained NN model. For a point $p_{channel}\left(s\right)$ on the reconstructed shape, the closest point on actual channel shape, $P_{p}\left(s\right)$ , was obtianed using *dist2curv* (D’Errico 2013). This point-to-curve distance is illustrated as the margenta color arrow in the Figure 8A inset. There are three types of the errors:

1) Average point-to-curve distance error: $\overset{L}{\underset{0}{\sum }}\left(||P_{p}\left(s\right)-p_{channel}\left(s\right)||\right)/L\;$

2) Distal tip distance error: $\left||P_{p}\left||(L\right)-p_{channel}\left(L\right)|\right|$

3) Accumulated angle error: $\overset{L}{\underset{0}{\int }}\kappa_{p}\left(s\right)ds-\;\overset{L}{\underset{0}{\int }}\kappa \left(s\right)ds$ .

The average point-to-curve distance error evaluates the difference between reconstructed shape and original shape. Larger these values are, more inaccurate the model is. As shown in Figure 8A inset, the distal tip distance error (black arrow) emphasizes the difference at the distal tip of the channel. This tip can be treated as the distal end of a continuum structure, where the end effector locates. C is the difference between accumulated angles, which directly affect the friction modeling of tendon sheath mechanism.

### One-Bend Shape (Dataset 1)

Table 3 presents the errors of the predicted results. For average point-to-curve distance error, the mean value on test set is 1.52 mm. The maximum value of distal tip distance error is 12.33 mm which is only 2.16% of the overall insertion length L . The maximum value of the accumulated angle error is 2.09 degree.

**TABLE 3:** Errors of prediction on test sets.

	Average point-to-curve distance error (mm)		Distal tip distance error (mm)		Accumulated angle error (degree)	
	Mean	Max	Mean	Max	Mean	Max
Dataset 1	1.52	4.19	5.15	12.33	1.13	2.09
Dataset 2	9.03	31.40	36.76	143.42	9.05	31.13

Figure 8 showcases some of the testing results. One-bend shape #54 had the maximum average point-to-curve distance error and #70 had the minimum average point-to-curve distance error. The predicted shapes in Figure 8A and the predicted curvatures in Figure 8B matched the actual shape well.

The 5-fold cross-validation results are presented in Table 4. Again, the training and testing scores are close to each other, indicating that the proposed network worked equally well on all data sets. Note that Dataset 1 contains only shapes with positive curvatures, and the network might have only learned the magnitude correlation between the insertion force and curvature.

**TABLE 4:** Training score and testing score of the Dataset 1

	Training score	Testing score
Split1	0.40	0.41
Split2	0.41	0.40
Split3	0.61	0.66
Split4	0.43	0.47
Split5	0.51	0.52
Mean	0.47	0.49
Standard deviation	0.08	0.10

**FIGURE 9:**
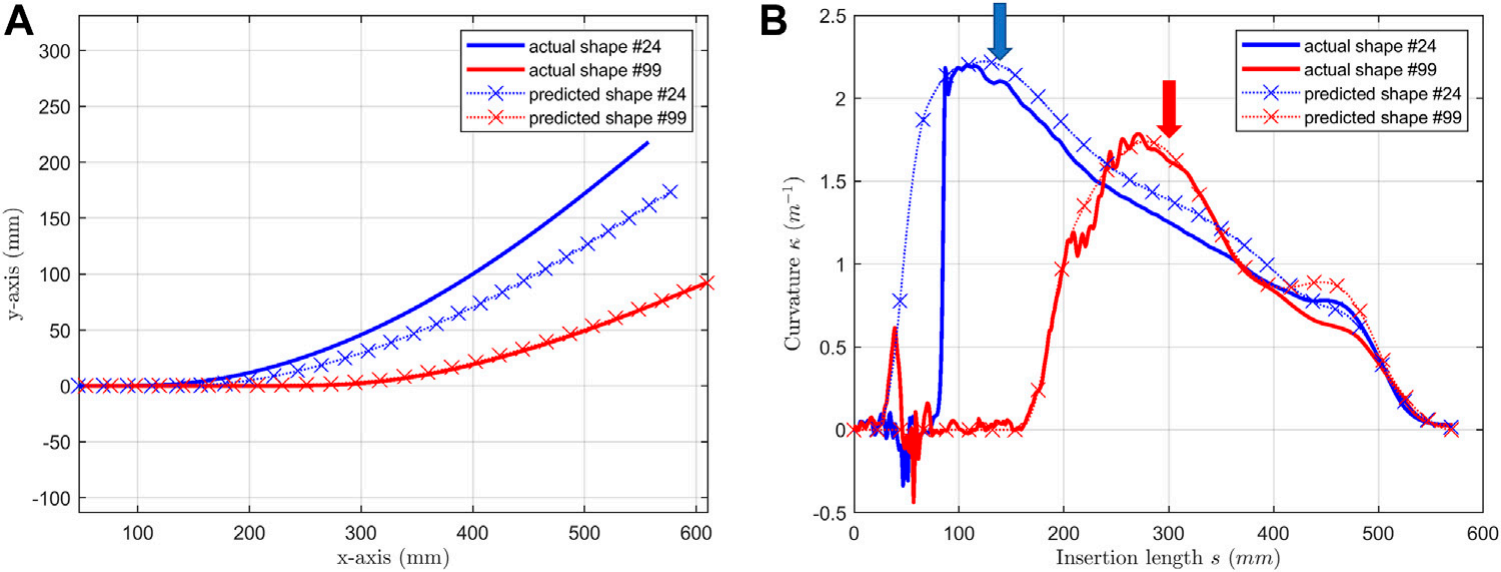
Selected shapes (shape #24 & #99) predicted by the NN model with unidirectional LSTM. **(A)** predicted shapes are compared with actual shapes; **(B)** predicted curvatures are compared with actual curvatures.

We also trained another neural network which has a two-layer unidirectional LSTM (forward only) with MLP of two hidden layers. Selected results Shape #24 and shape #99 are illustrated in Figure 9 for an example. It can be observed that the prediction before the peak is biased and fluctuated for shape #24 and #99 (the blue and red arrows). Furthermore, as the insertion length increases, the longer the length LSTM could use, the better the prediction is. However, the error accumulated in the beginning part of curvature prediction diverges the reconstruction based on it. The mean and max point-to-curve distance is 8.92 and 46.96 mm. The mean and max accumulated angle error is 3.09 and 7.15 degree. These errors are much larger than the proposed model.

Based on above results, we can summarize that the proposed model is good at learning the magnitude relationship between the insertion force and curvature.

### Two-Bend Shape (Dataset 2)

**FIGURE 10:**
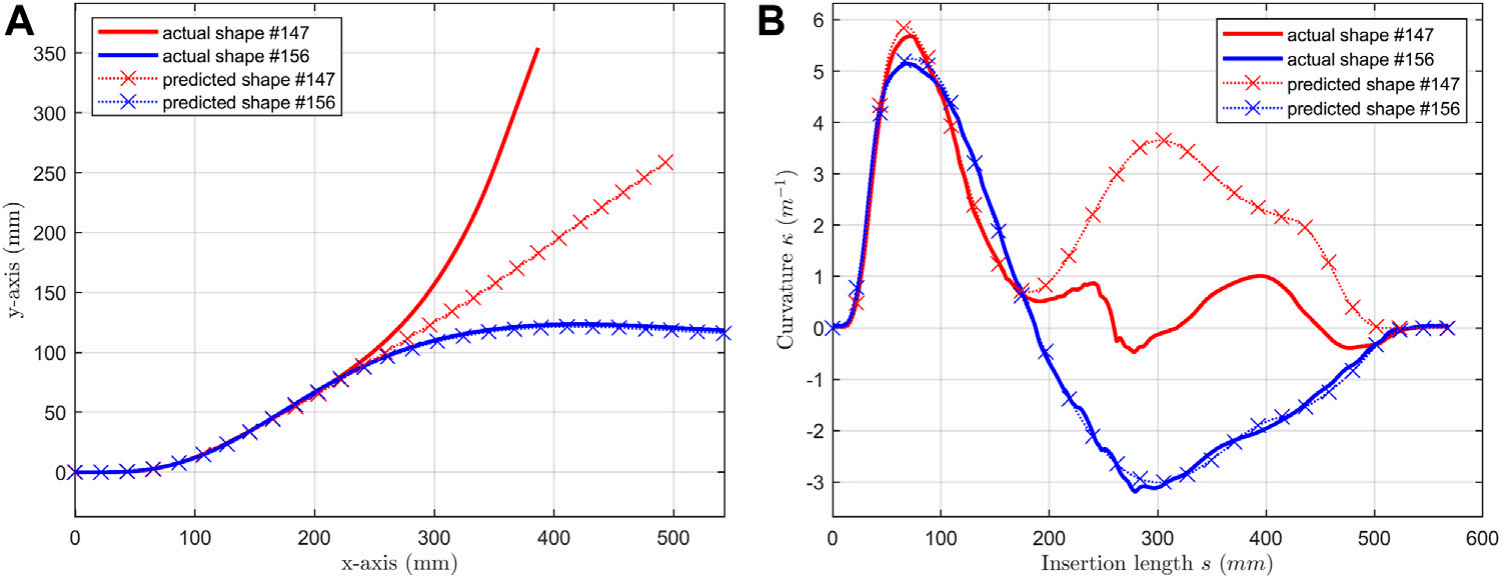
For shape #147 & #156, **(A)** predicted shapes are compared with actual shapes; **(B)** predicted curvatures are compared with actual curvatures.

The errors for two-bend shapes were huge, comparing with one-bend shape. The mean values for the three errors were: 9.03, 36.76 mm and 31.13 degree. We picked #147 and #156 as examples and illustrated in Figure 10. These two shapes had same first bend and opposite second bend. Shape #147 had two positive curvature bends and shape #156 had a positive bend followed by a negative bend.

Then, the predictions of shape #156 were accurate. The predictions of #147 were accurate only at the first bend section. However, in Dataset 2, 32 two-bend data were mixed with 120 one-bend data, which means the training data set was biased with positive curvature sample. The prediction should be accurate on #147 which had two positive curvature bends.

Thus, the correlation of magnitude learned from one-bend shapes could be learned and the curvature of the first bend could be accurately predicted. It also shows that the signs of curvatures cannot be predicted in this wok.

### Limitations of This Work

First, a ninth-order polynomial method is deployed for the curve fitting, which causes fluctuations at the channel’s straight part (a straight line). The fluctuation affects the curvature of tube shape calculation, which are labels feed to neural nets. At the same time, the insertion forces as training inputs are smooth and small over this range. This introduced defects in training process.

Second, we had unevenly distributed datasets. As introduced in Section *Experimental Setup*, the glass fiber reinforced plastic tube was deformed and fixed on the perforated board by cable ties to create the curved channel. The shapes formed depended on the location of the cable ties. The holes on the perforated board result in limited number of samples, and leading to uneven distribution of the curvature along the channel κ(s) . Moreover, for two bend shapes, we had few choices on the angle of first bend section and the curvature of second bend had different signs. These also led to an unevenly distributed dataset.

Third, the size of the data sets was small and the shapes were simple. We measured 120 one-bend shapes, with angles ranging from 0 to 87 degree and curvature ranging from 0 to 6.8 m^−1^. The range and samples were limited. The one-bend and two-bend shapes were really simple. Moreover, when we attempted the two-bend shapes, the shape of the second bend cannot be accurately predicted for all cases, which might be solved by increasing the amount and range of the data.

## Conclusion

We aimed to estimate the shape/curvature of a planar channel by the insertion force. A neural network model was developed to model the correlation based on the data collected on a testbed. The training results show the possibility of estimating the curvature magnitudes of the pathway based on the insertion force. However, predicting the signs of curvature is challenging. Future work is needed to further investigate the correlation between insertion forces and the pathway through mathematical models or finite element analysis and enhance the model’s capability in predicting more complex shapes. It is highly desirable to develop a simple formulation to theoretically or empirically model the correlation between insertion forces and curvature magnitudes. The approach may predict the curvature magnitudes and accumulated bending angles of flexible endoscopic surgical robots for accurate motion compensation and haptic force feedback.

## Data Availability

The raw data supporting the conclusion of this article will be made available by the authors, without undue reservation.
